# Health workforce burn-out

**DOI:** 10.2471/BLT.19.020919

**Published:** 2019-09-01

## Abstract

Increased demand for health services is putting unprecedented strain on health systems and the workers within them. Many are experiencing burn-out, depriving health systems of their most vital resource: people. Lynn Eaton reports.

For Dr Advik Gupta (name changed at his request) the crisis began with an overwhelming sense of futility. “I would get home to my wife and say I have achieved nothing today, even though I had been working flat out,” he says.

One of four consultant emergency physicians working in the emergency department of a district hospital in Cape Town, South Africa, Gupta was used to the life or death pressures of emergency care.

“The hospital is in an area notorious for its gang violence,” he says. “Around 4500 patients came through the department every month.” On a normal weekday roughly 30% of the patients were the victims of violence, but on the weekends the number and mix of cases changed. “On Friday nights there was a spike in penetrating trauma cases - mostly stab wounds and gunshot-related injuries.”

On such days as many as 80 acute trauma cases went through the department, overwhelming the hospital’s capacity to serve them.

Gupta is keen to emphasise that it was not the stress of dealing with so many acute trauma cases that made him feel like giving up, it was the chaos he experienced daily. As is often the case in health systems that lack effective primary care and referral systems, the emergency department had become the first port of call. Many of the less acute cases would have been better served by a primary health-care clinic or by social services.

“Burn-out is not just linked to the health of the doctor, it also affects the safety of the patient.”

Richard Heron.

Unable to cope, Gupta felt a strong sense of failure and shame. Like many people experiencing burn-out, he felt a compulsion to prove himself. “I tried harder,” he says. “Got in earlier, worked late.” It made no difference, and in the end, he decided to leave the hospital.

According to the World Health Organization’s (WHO) International Classification of Diseases (ICD), people experiencing burn-out typically feel exhaustion, but are also likely to feel detached or cynical about their job. They often perform less well at work, putting their patients at risk.

It is unclear how many people working in the health sector globally suffer from burn-out, as most research on this has been done in high-income countries. However, a recent survey by the International Occupational Medicine Society Collaborative, representing occupational medicine societies in 42 countries, provides some estimates.

The survey elicited responses on burn-out from health professionals from 30 countries across the income scale. Different comparability issues preclude drawing firm conclusions from the survey, but focusing solely on doctors reporting burn-out, the survey reported burn-out proportions ranging between 17.2% (Japan) and 32% (Canada), with Austria and Ireland reporting proportions comparable to those in Canada.

Dr Richard Heron, co-chair of the International Occupational Medicine Society Collaborative, draws attention to common drivers of burn-out, including excessive work-load and high patient expectations. These factors are complicated by an increased number of patients presenting with chronic diseases.

“There is an increased demand for services across the spectrum of health care, notably for the treatment of musculoskeletal, mental health conditions and other noncommunicable diseases such as cancer and heart disease,” Heron says. As people age, he adds, they are also more likely to be living with multiple, chronic diseases, imposing a demand for more complex treatments and integrated care.

At the same time, the promise of increased access to services in the context of universal health coverage, inevitably raises expectations.

For example, in 2009, China made a formal commitment to achieving universal health coverage for its 1.4 billion people and in the past 10 years health authorities have come close to achieving this goal with basic service coverage reaching more than 95% of its population.

Increased service provision has been matched by a sharp increase in outpatient and hospital admissions.

According to Professor Min Zhang, from the Chinese Academy of Medical Sciences, between 1995 and 2015, the number of outpatient visits in China increased by 100%. Admissions to public health institutions increased almost 300%. Meanwhile, the number of licensed physicians across China has increased by only 58%. China now has around 1.9 doctors per 1000 people compared to a high-income country average of 3.4.

“The disparity between capacity and demand has led to an overburdened workforce, increased waiting times and a lower quality of service than patients expect,” says Zhang.

Heron sees this dynamic expressed in many countries. “Quality suffers where staff are unable to cope,” he says. “The compassionate, caring environment is harder to maintain and mistakes are more likely. Burn-out is not just linked to the health of the doctor, it also affects the safety of the patient.”

In China and elsewhere, dissatisfied patients have attacked health workers. For Zhang, patient violence and health worker burnout are two sides of the same coin. “Burn-out contributes to workplace violence, then workplace violence contributes to burnout,” she says.

“There has been too much focus on the individual in addressing the burn-out problem.”

Advik Gupta.

Of course, the harms caused by burn-out are not limited to suboptimal care and patient dissatisfaction. Burn-out is also associated with increased absenteeism and turnover, which disrupts organizational function, reduces team efficiency and causes a loss of institutional knowledge.

What can be done to address this issue? Health professionals responding to the International Occupational Medicine Society Collaborative survey proposed interventions, such as improving work conditions and reducing or changing tasks. 

They also emphasized the need for monitoring, early diagnosis, and psychosocial risk factor prevention programmes.

Some low and middle-income countries are already implementing burn-out prevention or mitigation programmes. In Togo, for example, the Ministry of Health and Public Hygiene has been working with WHO and the International Labour Organization (ILO) on ways to avoid burn-out alongside other occupational hazards, such as infection risk and working in extreme heat. The psychosocial factors that might lead to burn-out are being assessed at 10 pilot sites.

“We have an occupational psychologist and an occupational nurse to help detect burn-out and build a strategy to prevent it,” says Dr Silvère Kevi, coordinator of occupational safety at the Togo health ministry. The project is just beginning and so it is too early to assess the impact.

In Sri Lanka, the Ministry of Health, Nutrition and Indigenous Medicine is starting with an occupational health, safety and wellbeing programme for health-care workers this year. 

According to Dr Inoka Suraweera, at the health ministry, it may be too soon to conclude that understaffing is the core burn-out issue. 

“Poor staffing levels may be responsible,” she says, but more evidence needs to be generated in this area, especially to support the planning of interventions. In my opinion, we need to study the effect of culture on this issue too, especially on coping.”

China has been collaborating with ILO and the China country office of WHO since 2013 on the use of a quality improvement tool for health facilities known as HealthWISE. The tool encourages managers and staff to work together to improve their workplaces and practices.

By dubbing burnout an ‘occupational phenomenon’ and defining it as a syndrome “resulting from chronic workplace stress that has not been successfully managed”, the ICD classification places as much emphasis on the workplace as the worker, suggesting that any meaningful response is going to require action on both sides of the equation.

Advik Gupta welcomes this approach. “Until now there has been too much focus on the individual in addressing the burn-out problem,” he says. “We need to see it from a health system point of view.”

Dr Ivan Ivanov, Team Lead, Global Occupational and Workplace Health at WHO headquarters, concurs, seeing occupational burnout as a symptom of poor working conditions in the health sector. 

“Ensuring decent working conditions in the health sector is a priority” he says, “and WHO and the ILO are working together to stimulate countries to develop national programmes for protecting the occupational health of health workers.”

**Figure:**
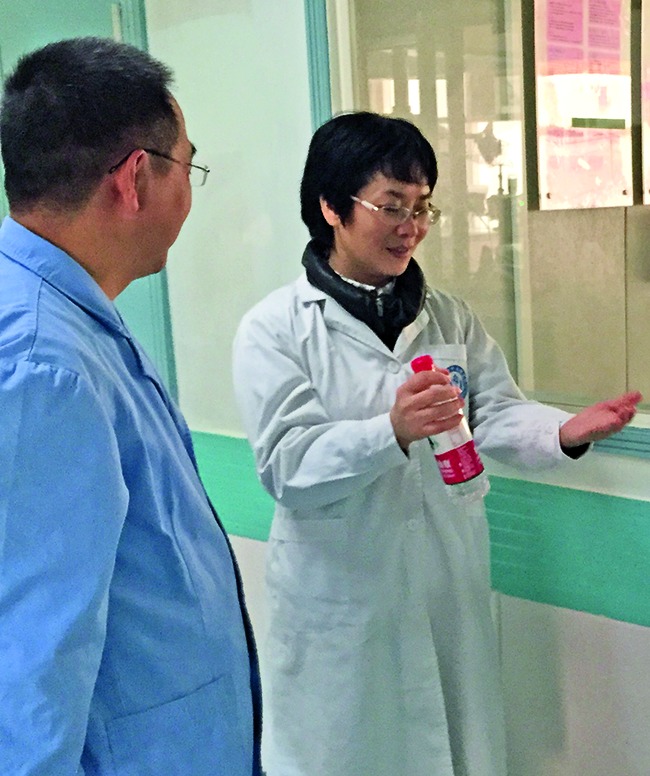
Professor Min Zhang discusses occupational health with infectious disease specialists at a hospital in Guangxi Zhuang Autonomous Region in China.

Courtesy of Min Zhang

**Figure:**
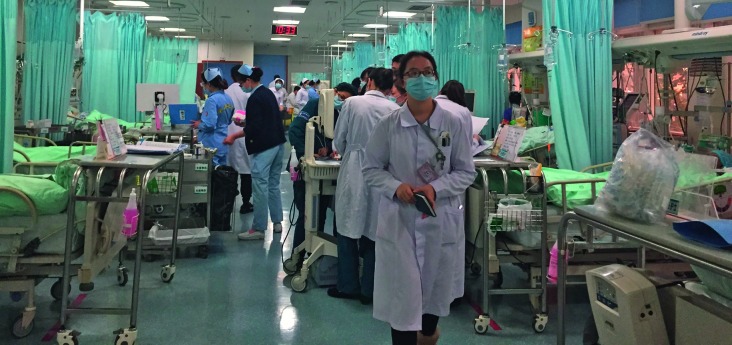
An emergency room of a tertiary hospital in central China's Henan province.

Courtesy of Min Zhang

