# Corrigendum

**DOI:** 10.2471/BLT.19.101019

**Published:** 2019-10-01

In: Health workforce burn out. Bull World Health Organ. 2019 Sep 1;97(9):585–86

on page 479, third column, the first sentence should read as follows:

“…(Canada), with Australia and Ireland reporting proportions comparable to those in Canada.”

